# Diagnosis of systemic lupus erythematosus by presence of Hargraves cells in eosinophilic pleural effusion: Case report

**DOI:** 10.1097/MD.0000000000012871

**Published:** 2018-10-19

**Authors:** Alexia D’Andréa, Damien L. Peillet, Christine Serratrice, Pierre-Augute Petignat, Virginie Prendki, Jean-Luc Reny, Jacques Serratrice

**Affiliations:** a Department of Internal Medicine, Rehabilitation and Geriatrics, University Hospital of Geneva, Trois-Chêne Hospital, Geneva; b Department of Internal Medecine, Hospital of Valais, Sion; c Department of Internal Medicine, University Hospital of Geneva, Geneva, Switzerland.

**Keywords:** eosinophilic pleural effusion, hargraves cells, LE cells, lupus erythematosus cells, systemic lupus erythematosus

## Abstract

**Rationale:**

Eosinophilic pleural effusion in elderly patients is most commonly due to malignancies and infections.

**Patient concerns:**

In rare cases, pleural eosinophilia is associated with connective tissue disease.

**Diagnoses:**

Presence of Hargraves cells, also called lupus erythematosus (LE) cells (polynuclear cells that have engulfed denatured nuclear material), was a key point of American College of Rheumatology (ACR) classification criteria for systemic lupus erythematosus (SLE) until 1997. Now replaced by serology for autoantibodies, LE cells characterization remains useful in guiding the diagnostic strategy towards autoimmune diseases.

**Interventions:**

An 82-year-old woman complained about anorexia, weight loss, fatigue, and mild night fever. Clinical examination disclosed a left pleural effusion without parenchymal lesion on high contrast thoraco-abdomino-pelvic computed tomography scan. A thoracocentesis revealed an exudate with eosinophilia. Direct cytological examination showed LE cells. SLE was rapidly considered. Antinuclear antibodies were subsequently found in the serum and in the pleural effusion. Anti-nucleosome antibodies were also present without antiphospholipid antibodies. Her condition rapidly improved after initiation of prednisone and hydroxychloroquine.

**Outcomes:**

Six months later, the patient had no particular complain, clinical examination was strictly normal biological parameter were in normal range.

**Lessons:**

The assessment of an eosinophilic pleural effusion allowed to find LE cells, which rapidly suggested the diagnosis of SLE, and early initiation of appropriate treatment. LE cells are no longer a criterion for the diagnosis of SLE, but their presence in serosa is most helpful in guiding the diagnostic strategy, and specifically in atypical forms often seen in older patients.

## 1 Introduction

In our observation, assessment of an eosinophilic pleural effusion allowed to find lupus erythematosus (LE) cells, which rapidly suggested the diagnosis of SLE in an elderly patient. Eosinophilic pleural effusion in elderly patients is most commonly due to malignancies and infections.^[1]^ In rare cases, pleural eosinophilia is associated with connective tissue disease. When associated with chronic joint pain and hematologic disorders, systemic lupus erythematosus (SLE) is part of the differential diagnosis.^[2]^ Discovered in 1946, the Hargraves cell is a macrophage or neutrophil that has phagocytized the nucleus of another cell.^[3]^ The formation of the LE cell or Hargraves cell is a reaction of the lupus factor with the nucleus of a normal polynuclear. The nucleus is phagocytized by another polynuclear and leads an LE cell.^[4]^ Despite a lack of sensitivity, presence of LE cells was a key point of American College of Rheumatology classification criteria for systemic lupus erythematosus until 1997.^[5]^ Now they were replaced by serology for autoantibodies.^[6]^ LE cells in pleural effusions are extremely rare. LE cells are easily detectable in seritis and remain useful to diagnose a systemic lupus erythematous in the older adults, with atypical clinical and serologic features.^[7]^ It's often difficult to consider a possible SLE in this population which is probably underdiagnosed. Our case recalls that this old test remains useful especially in older patients.

## 2 Case presentation

An 82-year-old woman with severe cognitive impairment (Mini Mental State score 14/30) was admitted in our department because of anorexia, weight loss, fatigue, and mild night fever. She complained of chronic knee and wrist pain. On clinical examination, she had a left pleural effusion without crackles or clinical signs of heart failure. There was no joint effusion, nor synovitis. Biological data were as follow: C-reactive protein: 59 mg/L (N < 5), White blood cell count: 3.3 G/L with 8.2% eosinophils, hemoglobin was 10 g/d L, platelet count was 150 G/L. A High contrast thoraco-abdomino-pelvic CT-scan showed multiple millimeter lymph nodes in the mediastinum, and a left pleural effusion without parenchymal lesion. A thoracocentesis (200 mL) revealed an exudate with 3065 M/L of predominantly lymphocyte-white blood cells (59%) with eosinophilia (20%). Direct cytological examination showed Hargraves cells, that is, LE cells, characterized by homogenous nuclear material (hematoxylin body) encompassed by neutrophils. No malignant cells were found, and pleural fluid culture was negative for Mycobacterium tuberculosis. A few days later, antinuclear antibodies (ANA) with homogeneous pattern were found with a titer of 2560 (N < 80) in the serum, and 5000 in the pleural effusion. Anti-nucleosome antibodies were also present (91 UI/L [N < 20]), without antiphospholipid antibodies. There was no hypocomplementemia. Search for HIV, CMV, EBV, HBV, HCV was negative, leading to the diagnosis of systemic lupus erythematosus according to ACR criteria. Hydroxychloroquine (400 mg/d) and prednisone 0.5 mg/kg/day with slow tappering, allowed patient recovery within 1 week, without recurrence of pleural effusion. Six months later, the patient had no particular complains, and clinical examination was strictly normal, C reactive protein was < 5 mg/L and ANA titer was 320. During the following year, she experienced no recurrence.

## 3 Discussion

SLE remains a challenging diagnosis in the older people as only 12% to 18% of the cases occur after the age of 50 years.^[7]^ Evoking the diagnosis is often difficult because of the lower incidence of discoid lupus, malar rash, or glomerulonephritis in the elderly, and arthritis, fever, serositis, lung disease, or neuropsychiatric symptoms can be easily misdiagnosed with more frequent pathologies. Pleural effusion is the most frequent finding in SLE.^[8]^ In our patient eosinophilia of the pleural effusion could have been misdiagnosed as the consequence of a malignancy of unknown origin, finding an high titer of ANA antibodies in the effusion, (beyond 160) was a strong argument in favour of its lupic origin.^[9]^ During SLE the effusion is usually made of lymphocytes and polynuclear cells. Eosinophilic cells are present only in rare cases.^[10]^

In our observation, assessment of an eosinophilic pleural effusion allowed to find Hargraves cells, which rapidly suggested the diagnosis of SLE in an elderly patient.

**Figure 1:**
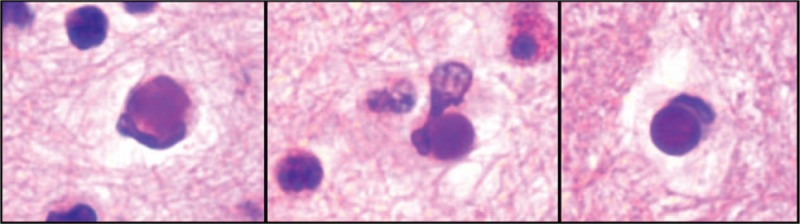
Hargraves Cells. Cellblock preparation of the pleural fluid showing several Hargraves cells with a dense cytoplasmic inclusion (hematoxylin body) (H&E, × 400).

Initially discovered by Malcolm Hargraves in bone marrow and peripheral blood, these cells have been since described in pleural, pericardic, synovial, peritoneal fluid.^[3,11]^ They can also be present in the cerebrospinal fluid and in the skin.^[12]^ These cells are either mononuclear cells, but more frequently neutrophils, that have engulfed denaturated nuclear material of another cell. They appear as a homogenous-looking mass, known as hematoxylin body, that compresses the nucleus of the phagocyte to the periphery of the cell^[13]^ (Fig. 1).

The exact pathophysiology of SLE remains unclear and is attributed to disruptions in adaptive immunity, triggered by genetic predisposing factors and various environmental insults. This disruption then leads to the loss of tolerance of self-antigens, involving T and B lymphocytes, with a key role of autoimmune reactivity.^[14]^ In the past decades, accumulating data support the major role of type-1 interferon pathway.^[15,16]^ More recently, the dysfunction of polynuclear has been emphasized, considering the potential central role of neutrophils extra cellular traps in the production of autologous double-standing DNA .^[17,18]^

Interestingly, decades after Hargraves cells were discovered, the dysfunction of polynuclear cells is increasingly thought to play a key role in the production of autologous double-standing DNA. Until 1997, the presence of LE cells was part of ACR criteria for the diagnosis of SLE.^[6,19]^ It was later discarded because of their lack of sensitivity and the concurrent improvement of indirect immune fluorescence. Search for Hargraves cells is less and less used in daily practice even if these cells are easily detectable in seritis; nevertheless, we argue that its characterization remains sometimes useful to the clinician in guiding the diagnostic strategy towards autoimmune diseases, and thus rapidly prompt an appropriate treatment, as in our observation. Assessment of an eosinophilic pleural effusion allowed to find Hargrave's cells, which rapidly suggested the diagnosis of SLE, and early initiation of appropriate treatment to control the disease and symptoms. If LE cells are no longer a criterion for the diagnosis of SLE, their presence in serosa is most helpful in guiding the diagnostic strategy, and specifically in atypical forms often seen in older patients.

## Author contributions

Alexia D’Andrea and Damien L. Peillet contributed in the study concept, data analysis, and interpretation, drafting the article.

Pierre-Auguste Petignat, Christine Serratrice, Jacques Serratrice, Virginie Prendki, and Jean-Luc Reny contributed in the data analysis and interpretation, and critical revision of article.

**Conceptualization:** Alexia D’Andrea, Damien L. Peillet.

**Supervision:** Christine Serratrice, Pierre-Auguste Petignat, Jean-Luc Reny, Virginie Prendki, Jacques Serratrice.
